# Management of Rarest Type of Talus Fracture: A Case Report

**DOI:** 10.7759/cureus.68830

**Published:** 2024-09-06

**Authors:** Aswin S, Madhan R, Dilip Kumar Naidu, Kevin Dhas, Gowthaman N

**Affiliations:** 1 Orthopedic Surgery, SRM Medical College Hospital and Research Centre, Chennai, IND

**Keywords:** a rare case, avascular necrosis of the talus, complex fracture, foot and ankle surgery, hawkins sign of talus, mini fragment plate and herbert screw for talus fracture fixation, osteochondral fragment, radiology finding, recent advance in orthopedic surgery, talus fracture

## Abstract

This is a case of the rarest type of talus fracture in a 28-year-old male who presented with pain in his right ankle and foot following a road traffic accident. He was unable to bear weight or walk after the injury. Imaging studies indicated fractures in the head and neck of the talus, as well as the talar dome, with a fracture line extending into the subtalar joint. The patient underwent open reduction and internal fixation using mini fragment plating and Herbert screw fixation for the osteochondral fragment. Both the intraoperative and postoperative periods were without complications. The patient was placed in plaster of Paris (POP) slab immobilization for four weeks and was advised to avoid weight-bearing while using a walker for eight weeks, after which physiotherapy commenced. Follow-up assessments showed satisfactory fracture union, good range of motion in the ankle, an excellent American Orthopedic Foot and Ankle Society (AOFAS) score, an excellent 17-Italian Foot Function Index (FFI) score, and a good Hawkins score.

## Introduction

Talus fractures occur mostly due to high-energy trauma. Although talus fracture incidence is low, it is the most fractured tarsal bone after the calcaneum. The talus has a major role in ankle and foot functions. The unique thing about the talus bone is that it has no muscle attachments [1]. The talus is covered by 60%-70% of articular cartilage. The primary blood supply to the talus comes from the tarsal canal artery, a branch of the posterior tibial artery that primarily nourishes the talar body. The talar neck is mainly supplied by the sinus tarsi artery, a branch of the perforating peroneal artery [2]. Extensive intraosseous anastomoses surrounding the talus contribute to its survival in severe injuries [3]. There are certain parameters that can significantly affect the talus blood supply, such as the timing of fracture reduction, the severity of damage to surrounding soft tissue, and the initial fracture displacement. The complications linked with talar neck and body fractures are avascular necrosis of the talus and osteoarthritis of the ankle joint, which is specifically related to the mode and injury severity of the talus, so these fractures should be diagnosed and addressed early to prevent complications and morbidity associated with them. Talar fractures were classified by various authors as follows: Canale and Kelly classification, Sneppen classification, the OTA classification, and the 2011 SOO "total talar fracture" classification [4-7].

## Case presentation

**Figure 1:**
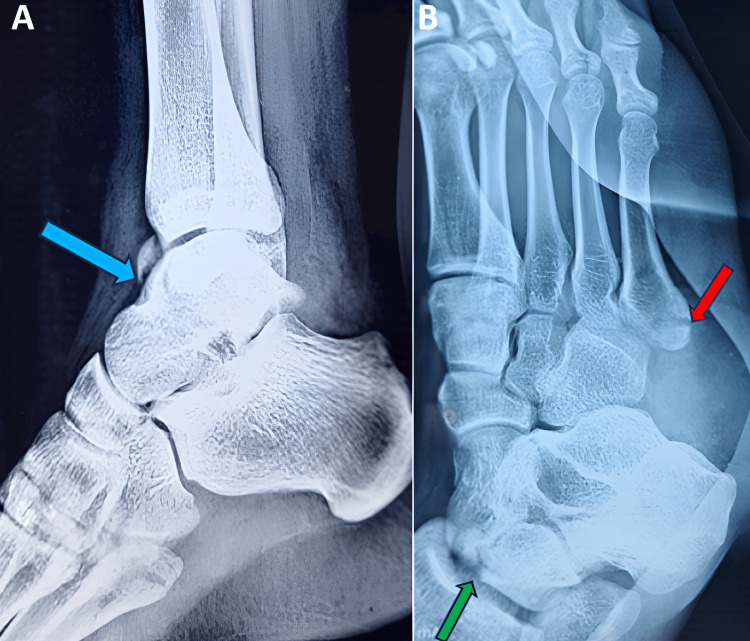
X-ray images of the right ankle and right foot. The images show (A) lateral view of the right ankle showing talus fracture (blue arrow) and (B) oblique view of the right foot showing fifth metatarsal base (zone 1) fracture (red arrow) and talus fracture (green arrow).

A 28-year-old male was admitted to the hospital with injuries to his right foot and right ankle. There was a reported history of road traffic accidents involving two-wheelers as opposed to four-wheelers. Pain in the right foot was sudden in onset, progressive, aggravating with ankle movements, and has no relieving factors. He was unable to do weight-bearing walking posttrauma. When examining the right foot as well as the right ankle, tenderness was elicited over the talus and fifth metatarsal base, and swelling was present over the dorsum of the midfoot, hindfoot, and ankle. The right foot and ankle range of movements were painful and restricted. There were no neurovascular deficits. The right leg was temporarily immobilized with a malleable splint. Radiological investigations were performed. A plain X-ray of the right ankle revealed a fracture of the neck and body of the talus (Figure 1A), and a plain X-ray of the right foot revealed a fracture at the base (zone 1) of the fifth metatarsal (Figure 1B).

**Figure 2:**
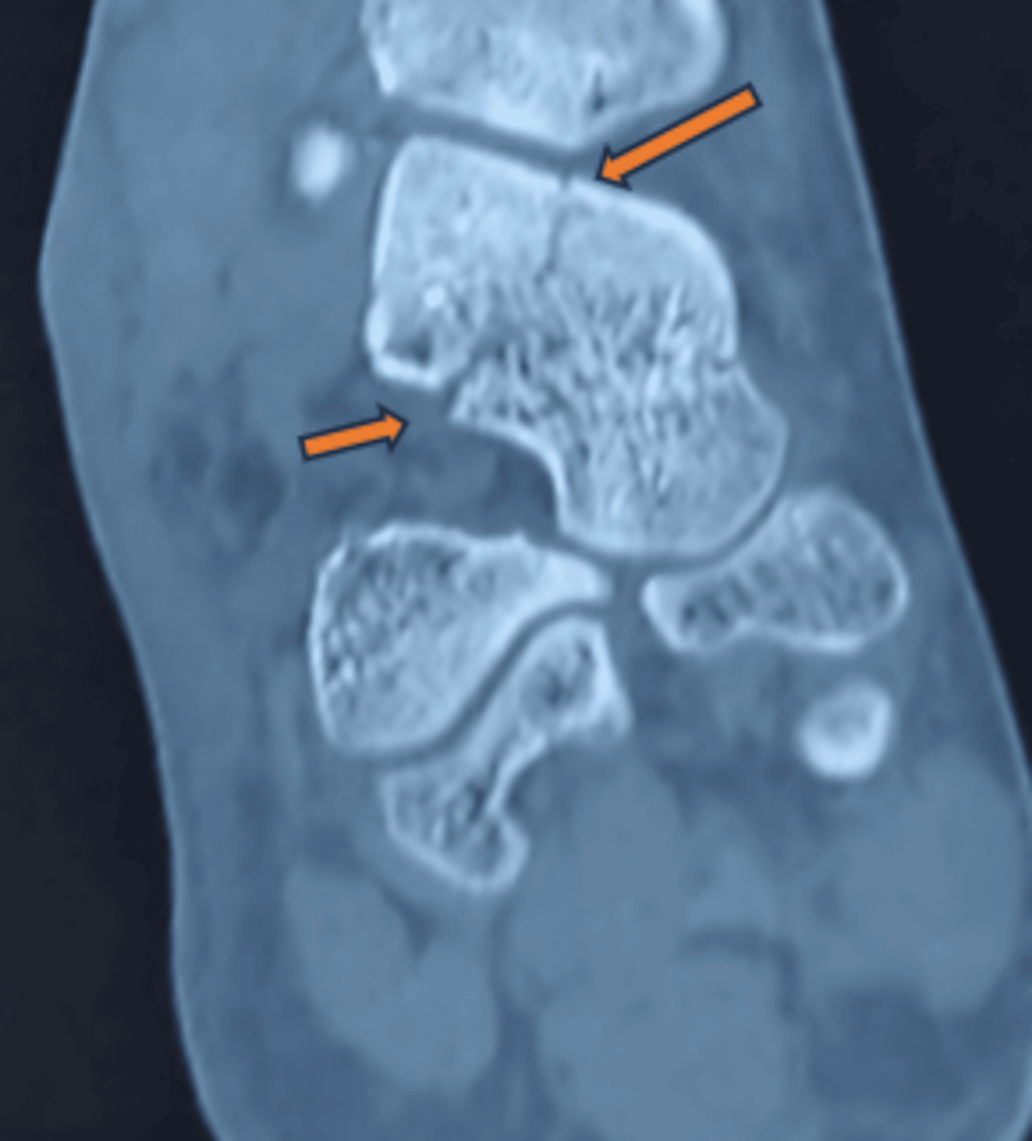
A CT scan of the right ankle coronal view reveals a fracture in the neck of the talus, which extends into the subtalar joint (orange arrow).

**Figure 3:**
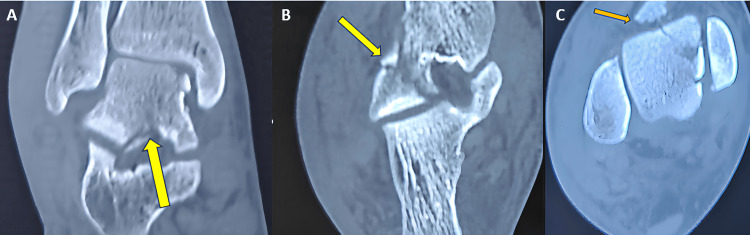
CT images of the right ankle. The images show (A) coronal view showing talus fracture extension into subtalar joint (yellow arrow), (B) axial view showing talus fracture extension into subtalar joint (yellow arrow), and (C) axial view showing talus fracture extension and fractured osteochondral fragment (yellow arrow).

**Figure 4:**
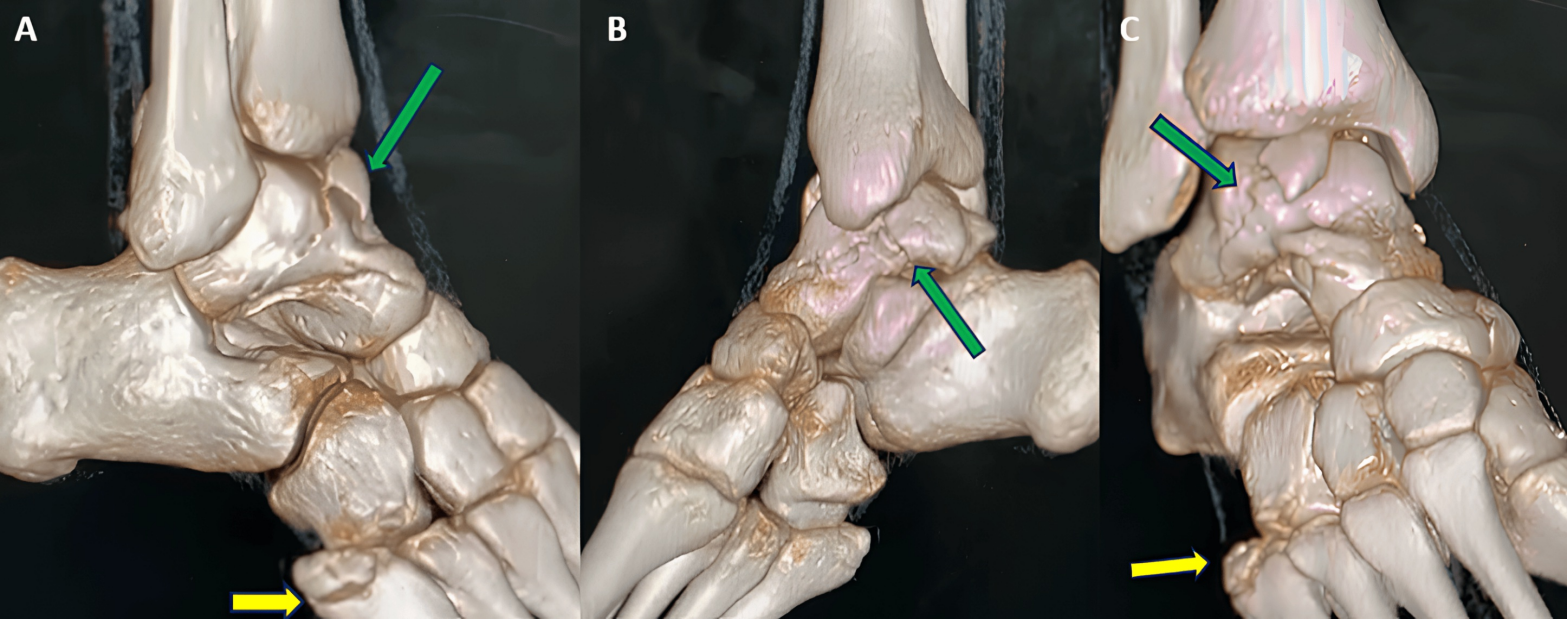
CT 3D images of the right ankle and right foot. The images show (A) lateral view of right ankle showing talus fracture extension into head, neck, subtalar joint, and displaced osteochondral fracture fragment (green arrow), fifth metatarsal base fracture (yellow arrow), (B) medial view of right ankle showing talus fracture extension into head, neck, subtalar joint (green arrow), fifth metatarsal base fracture (yellow arrow), and (C) anteroposterior view of right ankle showing talus fracture extension into head, dome, neck of talus and displaced osteochondral fracture fragment (green arrow), fifth metatarsal base fracture (yellow arrow).

Following a CT scan of the right ankle, it turned out that there was a talus fracture involving the head, neck, lateral process of the talus, talar dome (osteochondral fragment), and a fracture line that extended into the subtalar joint (Figures 2, 3A-3C, 4A-4C). Following that, the right leg was immobilized with a below-knee slab. Surgical management was planned based on CT findings. We have planned for open reduction and internal fixation of the talus fracture using a dual surgical approach (anteromedial and anterolateral approach) with chevron osteotomy of the medial malleolus for the talus fracture and conservative management for the fifth metatarsal base fracture.

Procedure

**Figure 5:**
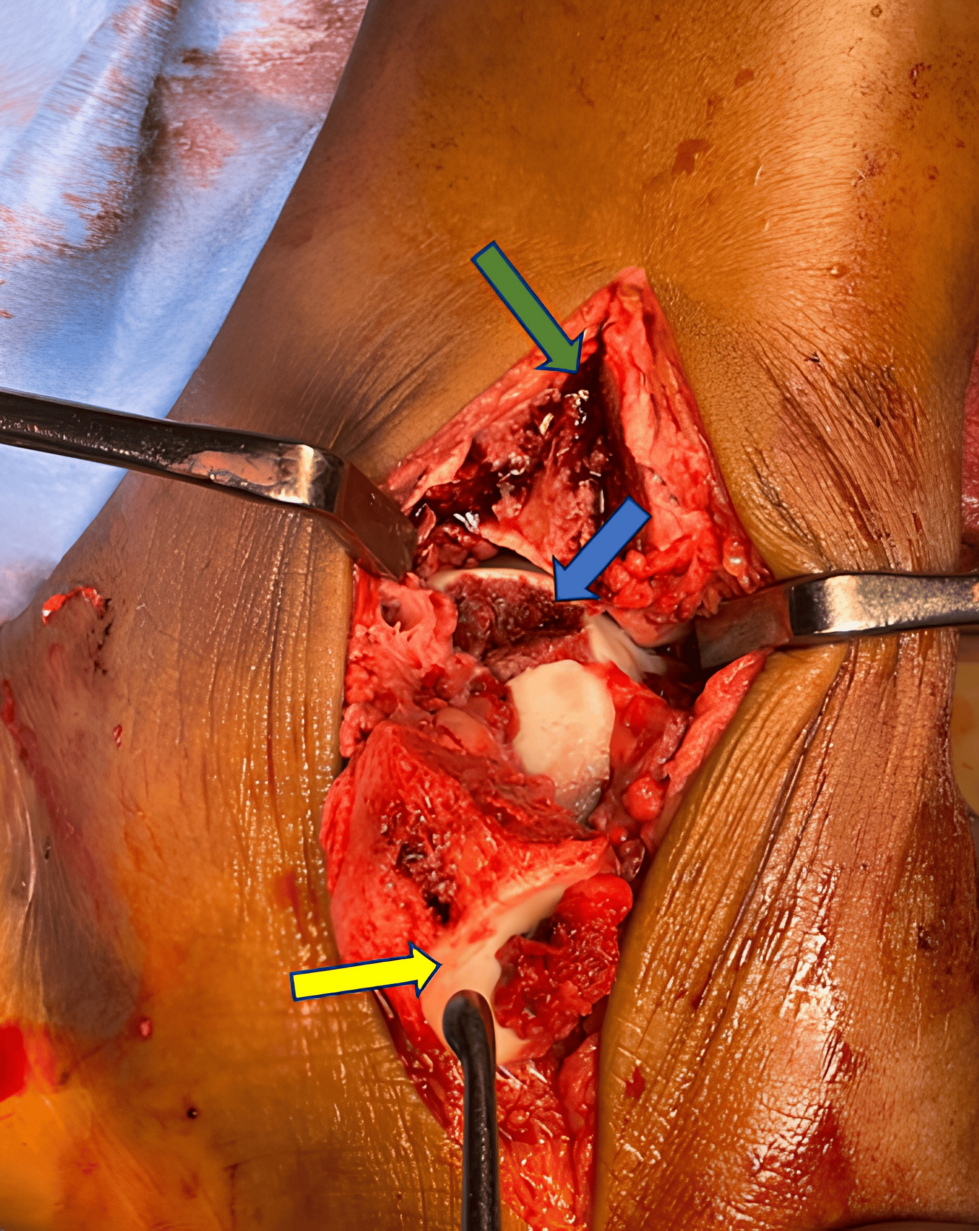
Intraoperative image of anteromedial approach. The images show an osteochondral defect at the dome of the talus (blue arrow), medial malleoli chevron osteotomy defect (green arrow), and medial malleoli osteotomized fragment (yellow arrow).

**Figure 6:**
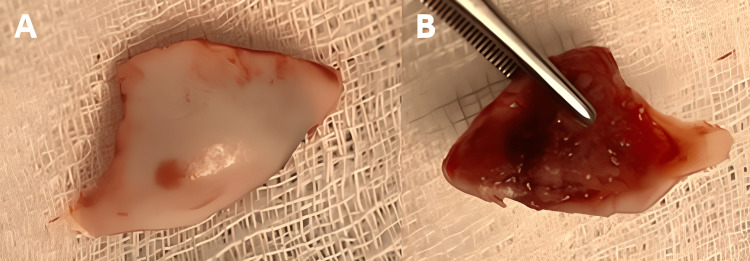
Intraoperative image of an osteochondral fracture fragment of the dome of the talus. The images show (A) superior view of a fractured osteochondral fragment of the talus (yellow arrow) and (B) inferior view of a fractured osteochondral fragment of the talus (yellow arrow).

**Figure 7:**
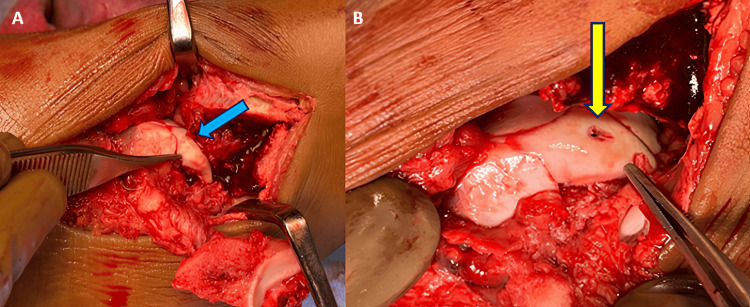
Intraoperative images of osteochondral fragment of talus fixation. The images show (A) a reduced fractured osteochondral fragment of the dome of the talus (blue arrow) and (B) a fractured osteochondral fragment of the talus fixed in anatomical position with Herbert screw (yellow arrow).

**Figure 8:**
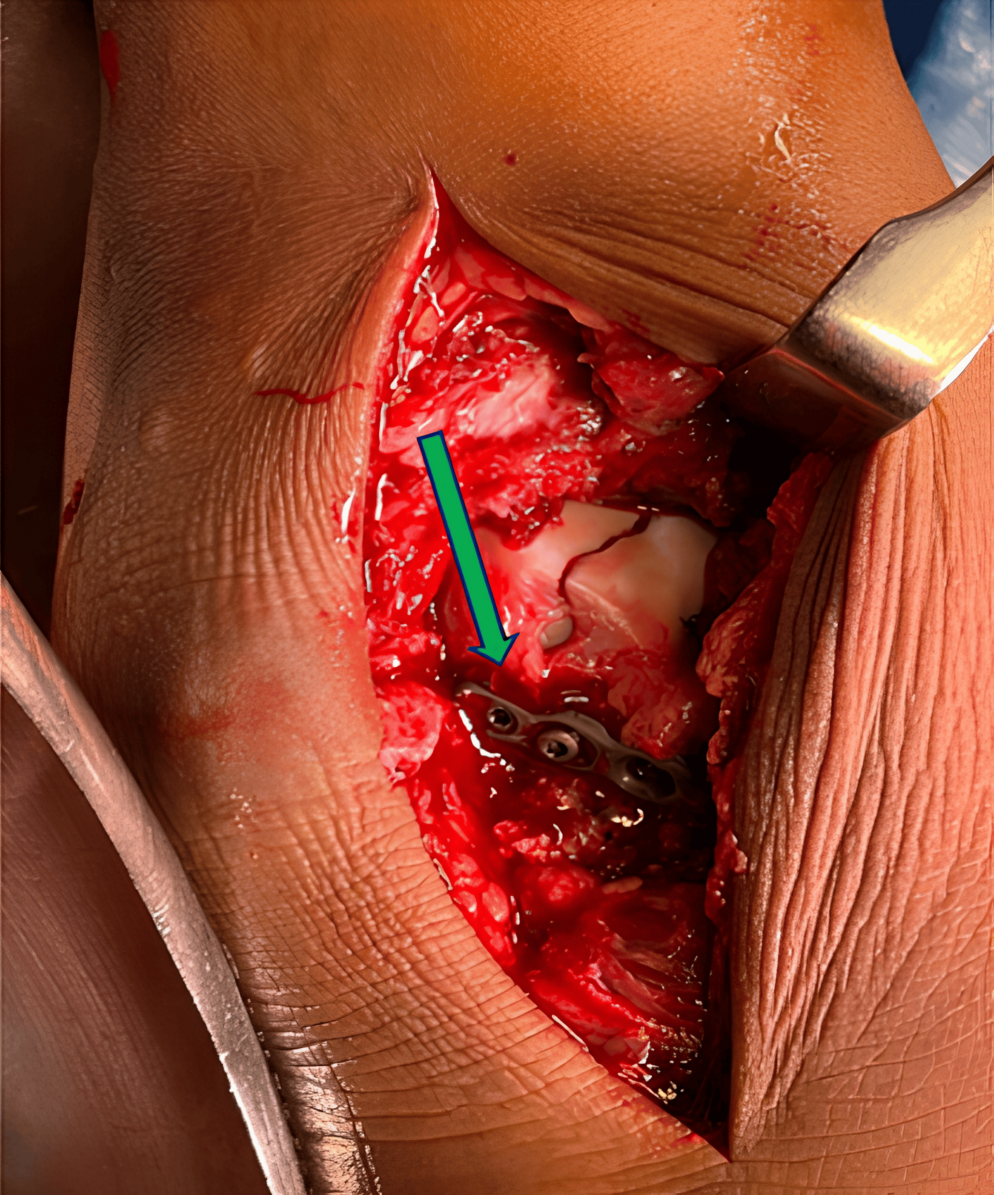
Intraoperative image of anterolateral approach. The image shows mini fragment plate fixation for talus fracture (green arrow).

Initially, we used the anteromedial approach. We made a 10 cm skin incision in the region between the tibialis anterior and posterior tendons, as well as on the medial malleolus. We then extended the incision towards the navicular tarsal bone along the medial border of the foot. To expose the medial malleolus without damaging the saphenous nerve and the great saphenous vein, we elevated the flap in tandem with the deep fascia. Next, we pre-drilled screw holes for the medial malleolus later fixation. A chevron osteotomy was carried out on the medial malleolus, and the osteotomized bone was folded along with the deltoid ligament (Figure 5). Afterward, we positioned the ankle in a varus position to clearly view the displaced osteochondral fractured fragment of the dome of the talus (Figures 6A, 6B). Then osteochondral fragment was reduced and kept in anatomical position by K-wire (Figure 7A), followed by definitive fixation with a Herbert screw (Figure 7B). Next, we secure the medial malleolus in place using two cannulated cancellous screws and a washer. The following anterolateral approach was taken. This required making a skin incision 10 cm proximally, centered between the tibia and fibula, and extending the incision distally across the ankle, centered over the fourth ray. We retracted the skin and subcutaneous tissue back in, ensuring the safety of the superficial peroneal nerve. We then performed a deep dissection, incising the fascia proximally, cutting the extensor retinaculum over the ankle, elevating the anterior compartment tendons, retracting them medially, and performing an arthrotomy of the ankle joint. We visualized and reduced the fractures in the head, neck, and body of the talus. We attached the fractured talus neck to the talus body with a 3.5 mm headless cannulated screw and then secured the head and body with a five-hole mini fragment locking plate (Figure 8).

**Figure 9:**
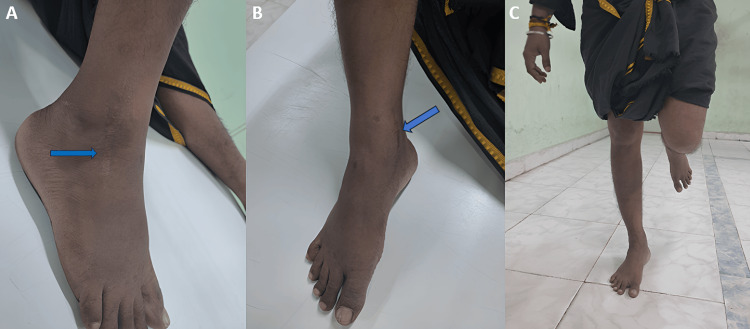
Clinical images on follow-up showing good functional outcomes postoperatively. The images show (A) healed scar of anterolateral approach surgical incision (blue arrow), (B) scar of anteromedial approach surgical incision (blue arrow), and (C) the patient's ability to do a single-leg weight-bearing stance without support.

**Figure 10:**
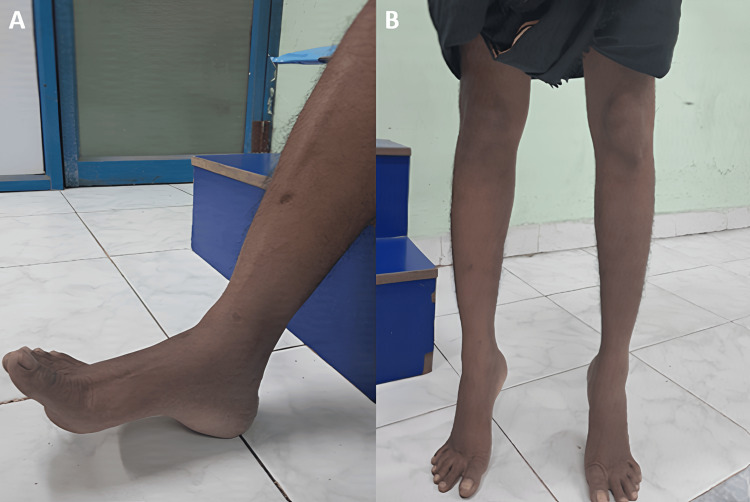
Postoperative images showing good range of movements on follow-up. The images show (A) good dorsiflexion and (B) good plantarflexion.

**Figure 11:**
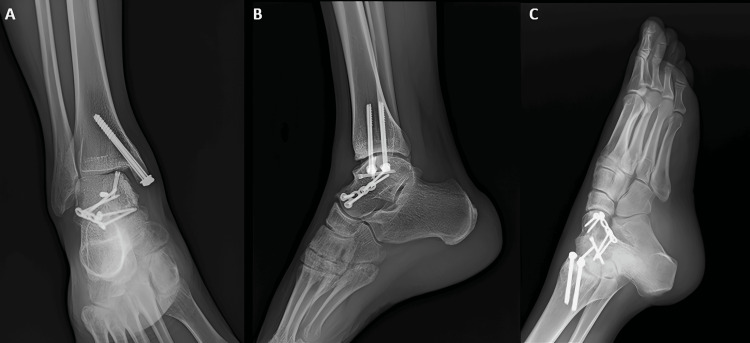
X-ray images of the right ankle. The images show (A) anteroposterior view on follow-up showing united talus fracture with no signs of avascular necrosis of talus and implant in position, (B) lateral view on follow-up showing united talus fracture with the implant in position, and (C) Canale and Kelly's view of the right ankle and foot on follow-up showing a united talus fracture and implant in position.

Under c-arm guidance, the fracture reduction and position were satisfactory. Next, we administered a thorough wound wash. The wound closed in layers. Distal pulses were felt. We then applied a sterile dressing and immobilized the ankle with a below-knee slab, keeping it in a neutral position. The operative period was uneventful. Postoperatively, we applied regular sterile dressing, and the wound healed well. The patient maintained a below-knee slab for eight weeks while engaging in non weight-bearing walking. After removing the below-knee slab, we initiated a period of partial weight-bearing walking. We also initiated and continued ankle and foot range-of-motion exercises for the next four weeks. Finally, we transitioned to full weight-bearing walking in the 12th week. On postsurgical follow-up, the patient showed excellent functional outcomes as per the American Orthopedic Foot and Ankle Society (AOFAS) score, 17-Italian Foot Function Index (FFI) score, a good functional outcome as per Hawkins score (Figures 9A-9C, 10A, 10B), and good radiological outcomes with no signs of avascular necrosis of talus (Figure 11A-11C). The patient was able to perform single-leg stance and weight-bearing walking without pain (Video 1).

**Video 1:** Follow-up video of the patient walking for assessment of functional outcome.

## Discussion

When used in isolation, the anteromedial or anterolateral technique has been linked to a malunion rate of 21.4%, attributed to insufficient fracture visibility [1,2]. The surgical technique employed must ensure sufficient access for fracture reduction to prevent malunion and devascularization of the talar neck. To prevent interruption of blood circulation during a dual-incision surgical procedure, it is important to preserve the deltoid ligament, sinus tarsi, and inferior talar neck. Additionally, there should be a sufficient skin bridge between these incisions and the elevation of full-thickness skin flaps [1,2]. Utilizing dual surgical approaches could successfully lower the incidence of avascular necrosis of the talus. Furthermore, this approach has been found to yield superior long-term clinical outcomes [2]. Analyzing talar fractures with a CT scan and using dual surgical approaches can enhance the outcome of the fracture [8]. This dual surgical approach improves the blood supply to the talus bone and ensures proper alignment of the fracture. Additionally, enhancing the internal fixation hardware, particularly the use of plates, is beneficial for treating complex talus fractures. The dual surgical approach also limits traction on bruised soft tissue [9-13].

In the present case, the talus fracture does not align precisely with the commonly used systems of classification. The fracture encompasses the head, neck, lateral processes of the talus, and talar dome (osteochondral fragment), with the fracture line extending into the subtalar joint. The intricate fracture pattern of the talus was managed using a dual surgical method. We performed a medial malleolar chevron osteotomy using the anteromedial approach, allowing for a wide and good exposure of the talar dome. The fractured osteochondral fragment was visualized, reduced, and fixed using a Herbert screw. Then the other fracture parts of the talus head, neck, and lateral process were addressed through anterolateral approach and internal fixation with mini fragment, and the postsurgical follow-up patient showed good functional outcomes and good radiological outcomes. The patient achieved an AOFAS score of 98 out of 100 [14], a visual analog scale (VAS) score of 0, a Hawkins score of 11 (good), and a 17-Italian FFI score of 0% [15].

## Conclusions

The atypical fracture pattern seen in this study posed a difficulty for both the surgeon and the radiologist in classifying it. This pattern was not classifiable as a whole in a single classification system. Thus, proper preoperative planning of the talus fracture is necessary for better reduction and fixation of the talus fracture. Adequate and wide exposure of talus without majorly compromising the blood supply to talus plays a vital role in definitive fixation. Early intervention for talus fractures is crucial to prevent the progression of untreated or inadequately reduced talus fractures, which can lead to avascular necrosis and posttraumatic ankle arthritis. In the present case, the follow-up showed a satisfactory radiological outcome with no avascular necrosis and a good functional outcome.
